# Dietary patterns and diabetic microvascular complications risk: a Mendelian randomization study of European ancestry

**DOI:** 10.3389/fnut.2024.1429603

**Published:** 2024-11-01

**Authors:** Xin Zhou, Wenbin Zheng, Wen Kong, Tianshu Zeng

**Affiliations:** ^1^Department of Endocrinology, Union Hospital, Tongji Medical College, Huazhong University of Science and Technology, Wuhan, Hubei, China; ^2^Hubei Provincial Clinical Research Center for Diabetes and Metabolic Disorders, Huazhong University of Science and Technology, Wuhan, Hubei, China; ^3^Hubei Key Laboratory of Metabolic Abnormalities and Vascular Aging, Huazhong University of Science and Technology, Wuhan, Hubei, China

**Keywords:** Mendelian randomization analysis, risk facors, casual effect, dietary patterns, diabetic microvascular complications

## Abstract

**Purpose:**

Previous observational studies about the link between dietary factors and diabetic microvascular complications (DMCs) is controversial. Thus, we systemically assessed the potential causal relationship between diet and DMCs risk using Mendelian randomization (MR) methods.

**Methods:**

We used genome-wide association studies (GWAS) statistics to estimate the causal effects of 17 dietary patterns on three common DMCs in European. Summary statistics on dietary intakes were obtained from the UK biobank, and data on DMCs [diabetic retinopathy (DR), diabetic nephropathy (DN), and diabetic neuropathy (DNP)] were obtained from the FinnGen Consortium. A two-sample MR (TSMR) was conducted to explore the causal relationships of dietary habits with DMCs. In addition, multivariable MR analysis (MVMR) was performed to adjust for traditional risk factors for eating habits, and evaluated the direct or indirect effects of diet on DMCs.

**Results:**

TSMR analysis revealed that salad/raw vegetable intake (odd ratio [OR]: 2.830; 95% confidence interval [CI]: 1.102–7.267; *p* = 0.0306) and fresh fruit intake (OR: 2.735; 95% CI: 1.622–4.611; *p* = 0.0002; false discovery rate [FDR] = 0.0082) increased the risk of DR, whereas cheese intake (OR: 0.742; 95% CI: 0.563–0.978; *p* = 0.0339) and cereal intake (OR: 0.658; 95% CI: 0.444–0.976; *p* = 0.0374) decreased the risk of DR. Salad/raw vegetable (OR: 6.540; 95% CI: 1.061–40.300; *p* = 0.0430) and fresh fruit consumption (OR: 3.573; 95% CI: 1.263–10.107; *p* = 0.0164) are risk factors for DN, while cereal consumption (OR: 0.380; 95% CI: 0.174–0.833; *p* = 0.0156) is the opposite. And genetically predicted higher pork intake increased the risk of DNP (OR: 160.971; 95% CI: 8.832–2933.974; *p* = 0.0006; FDR = 0.0153). The MVMR analysis revealed that cheese intake may act as an independent protective factor for DR development. Moreover, fresh fruit intake, salad/raw vegetable intake and pork intake may be independent risk factors for DR, DN and DNP, respectively. Other causal associations between dietary habits and DMCs risk may be mediated by intermediate factors.

**Conclusion:**

This causal relationship study supports that specific dietary interventions may reduce the risk of DMCs.

## Introduction

The global burden of diabetes mellitus (DM) is ascending mainly in response to economic development and lifestyle changes (1). According to reports, the global diabetes prevalence in those aged 20–79 years was estimated 536.6 million people, rising to 783.2 million in 2045 (2). Meanwhile, the incidence of diabetic microvascular complications also increased significantly (3). Diabetic microvascular complications (DMCs) with high impact on the quality of life and overall life expectancy mainly include diabetic retinopathy (DR), diabetic nephropathy (DN) and diabetic neuropathy (DNP). Approximately 25% of patients with DM suffer from DR, while DNP is encountered in nearly 50% of the diabetic population (4, 5).

DR is the primary cause of visual impairment and blindness in the working age population (6), which by 2045 affected more than 160 million individuals worldwide (7). While patients with DR may be asymptomatic in the early stage, it might rapidly progress into vision loss, visual field reduction, refractive changes, and reduced contrast sensitivity. At the end of the progression of DR, there will be neovascular proliferative membranes, traction retinal detachment, neovascular glaucoma, and eventually blindness, which place a considerable burden on patients’ quality of life (8). Despite efforts to find medical treatments to disease (8), still about a third of patients with DR suffer from severe non-proliferative DR or proliferative DR (PDR) or the presence of diabetic macular edema (DME) (9).

DN is the leading cause of end-stage renal disease (ESRD) worldwide. According to the World Health Organization (WHO), it is estimated that more than 200 million diabetic patients will develop into diabetic kidney disease (DKD) by 2045, which is an important cause of disability and death in patients with DM (10). DKD is characterized by deposition of extracellular matrix, thickening of the glomerular basement membrane, altered proliferation and tubular atrophy, leading to interstitial fibrosis and glomerulosclerosis, and eventually renal failure (11). Although the field of DN has made great progress, the number of diabetic patients with ESRD continues to increase (12).

DNP can involve both the central and peripheral nerves, the latter being particularly common (13). The main clinical manifestations are pain, loss of limb sensation, falls, and an increased risk of foot ulcers and lower limb amputations (14). People with prediabetes also develop peripheral neuropathy, which becomes more severe after the transition to a pronounced DM (15). Severe neuralgia affects the quality of life of people with diabetes, including limited activity, depression, and impaired social functioning (16).

Therefore, finding effective ways to prevent or control of DMCs are of critical importance. The control of blood glucose, blood pressure and blood lipids are common risk factors for the development of DMCs. Aside from these factors, it has been proposed that certain food intake may modify the risk of these complications (17–19) through its impact on gut microbes and inflammation, which are critical factor in pathogenesis of DM and related diseases (20–22). A longitudinal study showed that the Mediterranean diet pattern is linked to a lower risk of DMCs among a cohort of patients with DM in Iran (23). However, a *Post Hoc* Analysis of a randomized controlled trial (RCT) concluded that a Mediterranean diet may not protect against diabetic nephropathy (24). In addition, a cross-sectional study by Lee and associates found that coffee consumption reduced the prevalence of DR in diabetic patients younger than 65 years of age in Korea (25), whereas Kumari and associates found no significant association between coffee and DR in their cross-sectional study (9).

Given the inconsistent results of previous studies, the effect of food patterns on different DMCs needs to be investigated. It has been a challenge to investigate the causality of diseases by lifestyles using observational studies or RCTs due to ethical constraints and technical issues such as potential residual confounders and reverse causation bias (26). Mendelian randomization (MR) analysis helps to avoid these limitations owing to the unique properties of genotype (27). In MR Analyses, single nucleotide polymorphisms (SNPs) robustly related to an exposure are used as instrumental variables (IVs) to investigate causal association between exposures (food patterns) and outcomes (DMCs) (28). In the absence of RCTs, MR analysis is a vital strategy for causal inference as genetic variants are randomly assorted at meiosis, in which the process mimics an RCT.

To date, only one study using the MR to estimate the associations of genetically predicted coffee consumption in relation to DN (10). Hence, to thoroughly disentangle the causal relationship between dietary habits and the risk of DMCs, we applied two-sample MR (TSMR) and multivariate MR (MVMR) approaches to determine the direct causal effects of dietary habits on DMCs and also performed sensitivity analyses to ensure the robustness of the results. The study may be helpful to provide scientific strategies for primary prevention of DMCs.

## Materials and methods

### Study design and data sources

**Figure 1:**
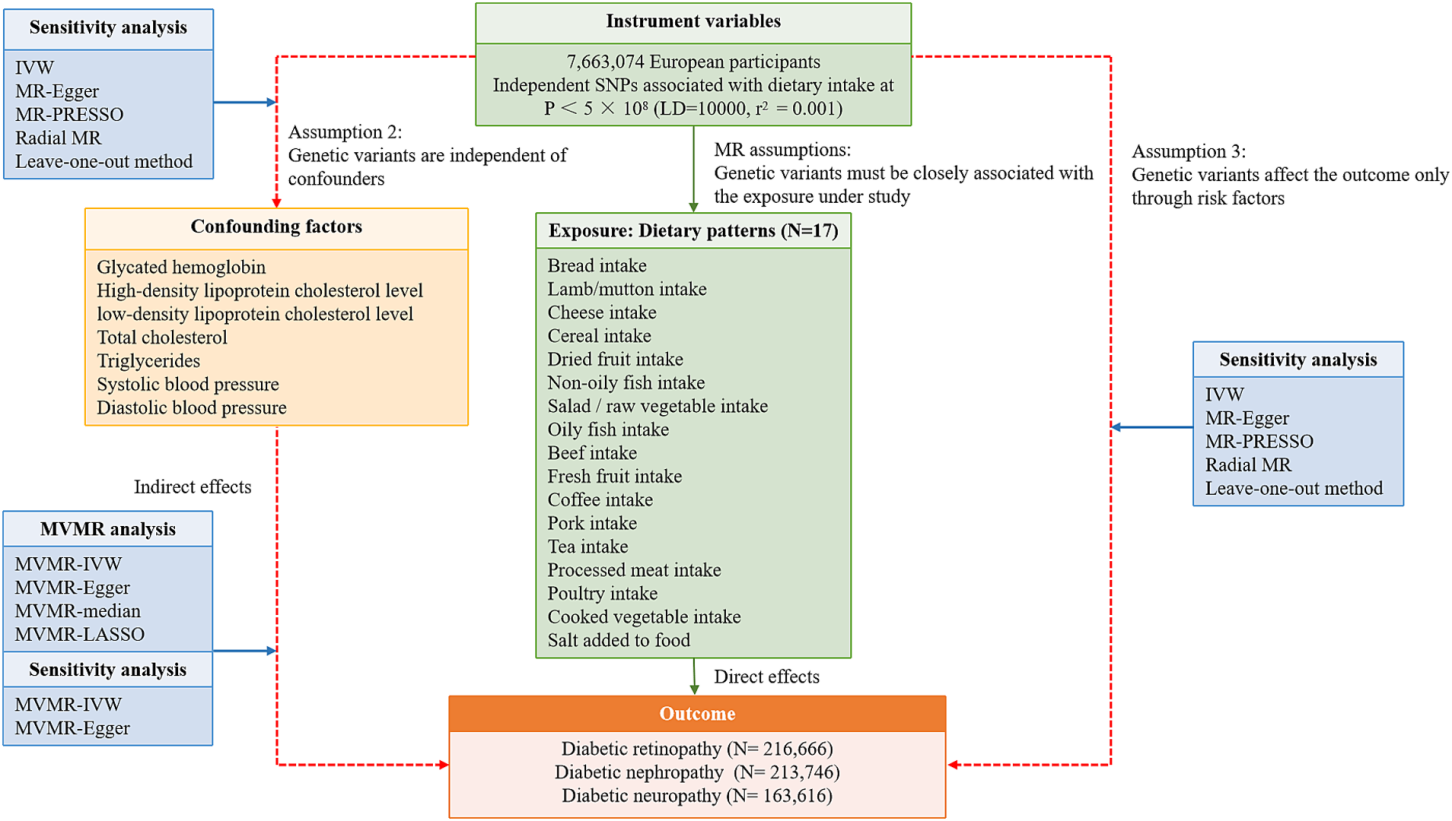
A flowchart describes the study design briefly. SNP, single nucleotide polymorphism; LD, linkage disequilibrium; MVMR, multivariable Mendelian randomization; IVW, inverse variance weighted.

A flowchart describes the study design briefly (Figure 1). The MR design relies on three core assumptions: (1) Genetic variants must be closely associated with risk factors; (2) Instrumental variable (IVs) are independent of confounders; (3) IVs affect the outcome only through risk factors. The dataset used in this study was retrieved from public databases and ethical approval was obtained prior to implementation. The datasets used in our study are retrieved from the reanalysis of previously summarized data. Therefore, this study does not need additional ethical approval. The Strengthening the Reporting of Observational Studies in Epidemiology using Mendelian Randomization (STROBE-MR) checklist for this study is presented in Supplementary Table S1. TSMR approach was employed to evaluate the causal effects of 17 dietary habits on the occurrence of three common DMCs. The multivariable adjustment was performed by including traditional risk factors for DMCs that were positively associated with the outcome risk.

In this study, the summary statistical data of 17 dietary patterns was obtained from the UK Biobank (UKB) cohort, available on the Integrative Epidemiology Unit (IEU) Open GWAS project.^1^ The exposures included meat intake (Lamb/mutton intake, Beef intake, Pork intake, Poultry intake, Oily fish intake, Non-oily fish intake and Processed meat intake), vegetable intake (Cooked vegetable intake and Salad/raw vegetable intake), fruit intake (Dried fruit intake and Fresh fruit intake), staple food intake (Cereal intake and Bread intake), beverage intake (Coffee intake and Tea intake), and fermented milk intake (Cheese intake) and another food intake (Salt added to food). Data on the dietary patterns were gathered using a retrospective dietary frequency questionnaire that can be accessed through the UKB.^2^ If the responses provided by the participants were deemed implausible, the questionnaires were not accepted. Further details regarding the dietary questionnaires are outlined in Supplementary Table S2.

The GWAS summary statistics for DMCs were extracted from the FinnGen consortium (29).^3^ The GWAS for DR, DN, and DNP included 14,584 cases and 202,082 controls, 3,283 cases and 210,463 controls, 1,415 cases and 162,201 controls, respectively, all of European ancestry.

Previous observational studies have suggested that glycemia, blood pressure and blood lipid are important risk factors for the progression of DMCs (30, 31). Therefore, in our MVMR analysis, we included key risk factors identified in the TSMR, as well as conventional risk factors, aiming to identify independent risk factors influencing DMCs. The summary-level genetic information for glycated hemoglobin, which could reflect the average blood glucose level over the last 3 months, was retrieved from a study published recently (32). For the blood pressure dataset, the summary data for both systolic blood pressure (SBP) and diastolic blood pressure (DBP), from the International Consortium of Blood Pressure (ICBP), was extracted from IEU Open GWAS project (33). Routine blood lipid tests include total cholesterol (TG), triglyceride (TG), low-density lipoprotein cholesterol (LDL-C), and high-density lipoprotein cholesterol (HDL-C). The GWAS summary data for the aforementioned four lipids were also extracted from IEU Open GWAS project (34). The detailed sources of summary-level data are displayed in Supplementary Table S3.

### Selection criteria for IVs

The selection of IVs met the following requirements: (i) SNPs significantly associated with dietary patterns were extracted from the GWAS summary statistics (*p* < 5 × 10^−8^); (ii) to ensure independence between SNPs, the linkage disequilibrium (LD) parameter condition were set at *r*^2^ = 0.001, with a cluster window of 10,000 kb; (iii) The strength of each single IV was quantified with an F-statistic calculated as *β*^2^/se^2^ (*β*: estimated effect of SNP; se: standard error of the genetic effect), and weak IVs with an F-statistic <10 were excluded; (iv) We excluded palindromic SNPs with intermediate allele frequencies, SNPs associated with outcomes (*p* < 5 × 10^−5^) and SNPs with minor allele frequency (MAF) less than 0.01.

### Statistical analysis

For TSMR analysis, Five methods [inverse-variance weighted (IVW), MR Egger, weighted median, weighted mode methods, and Bayesian weighted Mendelian randomization (BWMR)] were performed to examine causality association between food intakes and different diabetic complications, with IVW method being the primary MR analysis and the others being supplementary analyses (35). Effect estimates from MR analyses were reported as odds ratios (ORs) with corresponding 95% confidence intervals (CIs). IVW calculates the effect of genetic variation on exposure and outcome by means of the ratio method and is applicable in the absence of pleiotropy (36). MR-Egger regression analysis can still estimate the causal effect of the outcome if the included SNPs are pleiotropic (37). The weighted median method requires that at least 50% of the SNPs meet the premise of being valid instrumental variables. After the included SNPs are arranged according to the weight, the median of the relative distribution function is obtained as the analysis result (38). BWMR is able to accounts for the uncertainty of estimated weak effects and weak horizontal pleiotropic effects, as well as adaptively detect outliers due to a few large horizontal pleiotropic effects (39). Consistent causal effects of the exposures on outcomes may be more robust among several approaches (40). Moreover, IVW and MR-Egger regression were used to test for heterogeneity, quantified as Cochran’ s Q statistic (37, 41). If a *p*-value less than 0.05 indicated heterogeneity, an IVW random effects model was used to mitigate potential effects. In contrast, if the p-value >0.05, there was no heterogeneity and a fixed effects model was used (42). The MR-Egger intercept method calculated the intercept after the linear regression analysis to assess horizontal pleiotropy (43). MR pleiotropy residual sum and outlier (MR-PRESSO) and radial MR tests were employed to identify and check for outliers and horizontal pleiotropy (44). If potential outliers were found, the TSMR analysis was performed again after removing them. Finally, the leave-one-out method was conducted to identify the stability of results. In addition, the Benjamini–Hochberg method was used for the false discovery rate (FDR) correction, with the threshold set as 5% FDR, to correct for multiple comparisons. We reduced the likelihood of incorrectly identifying significant outcomes due to chance fluctuations in multiple comparisons, thereby improving the dependability of our results (45).

For MVMR, the methods MVMR-IVW, MVMR-Egger and MVMR-Median were used to evaluate the independent association between each exposure and outcome, with MVMR-IVW as the main analysis method (46). The MVMR results were re-adjusted using the least absolute shrinkage and selection operator (LASSO) method. MVMR-IVW and MVMR-Egger were used to detect heterogeneity. When heterogeneity was detected, the MVMR-median model was prior to the MVMR-IVW model as it naturally accounts for heterogeneity via a bootstrapped variance (47). MVMR-Egger intercept test was performed to determine the possible pleiotropy, with the intercept *p*-value being less than 0.05, indicating considerable horizontal pleiotropy (48).

Statistical analyses were performed by R software (version 4.3.2) with the following packages: “TwoSample MR package” (0.5.6), “MendelianRandomization” (0.6.0), “MVMR package” (0.3.0), “BWMR” (0.1.1), “MR-PRESSO” package and “RadialMR” package (version 1.0).

## Results

### TSMR analyzes the potential impact of 17 dietary habits on three diabetic microvascular complications

The publicly available meta-analyses of 17 dietary patterns and three types of DMCs were extracted from MRC-IEU and FinnGen Biobank, respectively, with little population overlap between exposures and outcomes. In total, 423 SNPs, robustly and independently associated with the 17 dietary habits, were used in this study after a series of quality control steps. The number of SNPs of each exposure ranged from 5 to 76, as detailed in the Supplementary Tables S4–S6. All F-statistics were greater than 10 (range: 10.067–342.831), meaning that the analysis results are not biased by the influence of weak IV.

**Figure 2:**
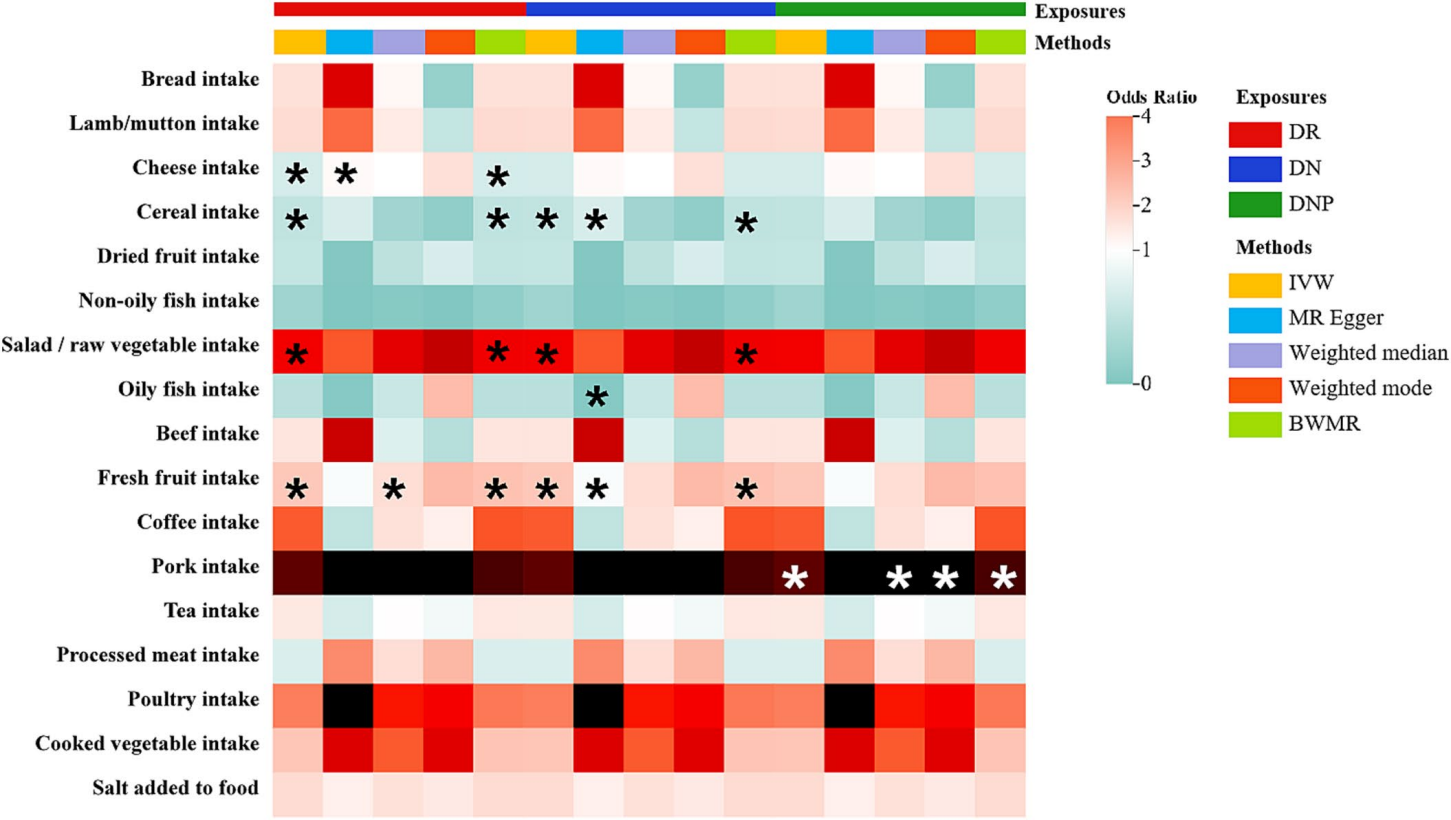
The heatmap illustrates the causal relationships between for various dietary intakes and three common diabetic microvascular complications (DMCs). Total effect sizes for associations between dietary intakes and DMCs were estimated using five different methods. Asterisks indicate that the association is nominally significant (*p* < 0.05). Color is scaled based on the Mendelian randomization odds ratio estimates, with green indicating protective factors and red risk factors.

In the TSMR analyses, a total of four associations from 17 dietary habits to DR were identified (*p*<0.05 by IVW method). And three causalities were found for DN, while only one causal relationship was observed for DNP (Figure 2). Complete results of the association between 17 eating habits and three common types of DMCs are summarized in Supplementary Tables S7–S9. As shown in Figure 2, we found evidence that higher intake level of salad/raw vegetable (OR: 2.830; 95% CI: 1.102–7.267; *p* = 0.0306) and fresh fruit (OR: 2.735; 95% CI: 1.622–4.611; *p* = 0.0002; FDR = 0.0082) were associated with increased risk of DR. On the contrary, genetic predisposition to increased consumption of cheese (OR: 0.742; 95% CI: 0.563–0.978; *p* = 0.0339) and cereal (OR: 0.658; 95% CI: 0.444–0.976; *p* = 0.0374) were protective against. And we have reached the same conclusion in the BWMR method. For DN, genetically predicted that salad/raw vegetable (OR: 6.540; 95% CI: 1.061–40.300; *p* = 0.0430) and fresh fruit (OR: 3.573; 95% CI: 1.263–10.107; *p* = 0.0164) intake frequency was associated with an increased risk of DN, which was identified by BWMR method (OR: 6.881; 95% CI: 1.058–44.762; *p* = 0.0435; OR: 3.790; 95% CI: 1.298–11.066; *p* = 0.0148, respectively). However, cereal consumption (OR: 0.380; 95% CI: 0.174–0.833; *p* = 0.0156) was associated with a decreased risk of DN, which was also identified by BWMR and Weighted median method. For DNP, genetically driven pork intake was related to an increased risk of DNP (OR: 160.971; 95% CI: 8.832–2933.974; *p* = 0.0006; FDR = 0.0153), which was further verified by the other three methods (Weighted median: OR: 383.970; 95% CI: 9.020–16345.129; *p* = 0.0019; Weighted mode: OR: 763.460; 95% CI: 3.097–188197.993; *p* = 0.0458; BWMR: OR: 183.341; 95% CI: 8.610–3904.028; *p* = 0.0008).

MR-Egger regression and IVW analysis, used to detect heterogeneity of all results, did not show heterogeneity (Supplementary Tables S10–S12). Similarly, the MR-Egger intercept did not show horizontal pleiotropy (Supplementary Tables S13–S15). In addition, the funnel plots showed symmetry, suggesting a balanced pleiotropy (Supplementary Figures S1A–H). Leave-one-out results demonstrated that no individual SNP could significantly affect the causal estimation (Supplementary Figures S2A–H). Taking all these results into consideration, we could conclude that the Univariable MR (UVMR) results were robust and had limited bias.

### Multiple variables Mendelian randomization

**Figure 3:**
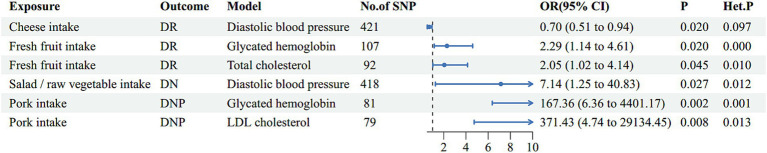
The multivariate analysis results of risk factor diets with traditional risk factor adjustment. Het. P refers to *p*-values for heterogeneity of inverse variance weighted method and Het. P < 0.05 indicates potential heterogeneity, for which case the weighted median method was utilized for causal inference. DR, diabetic retinopathy; DN, diabetic nephropathy; DNP, diabetic neuropathy; SNP, single nucleotide polymorphism; OR, odds ratio; CI, confidence interval.

To determine whether these above associations were direct risk factors for DMCs occurrence or mediated through traditional risk factors (glycemia, blood pressure and blood lipid), we performed MVMR analyses. Figure 3 and Supplementary Tables S16–S19 present the MVMR analysis results. In MVMR analysis controlling DBP as a covariate, robust evidence was demonstrated for a direct causal effect of cheese intake on the protection of DR. However, after adjusting for other confounding factors, the estimated MR value of cheese intake lost its statistical significance or observed horizontal pleiotropy, suggesting that these factors may partially mediate the effect of cheese intake on DR. And after adjusting for glycated hemoglobin or TC individually, we still observed an increased risk for DR with fresh fruit consumption. What’s more, the association between salad/raw vegetable intake and DN remained significant even after incorporating DBP into the multivariate models. Nonetheless, the associations of cereal or salad/raw vegetable consumption with DR and cereal or fresh fruit consumption with DN were non-significant after adjustment for traditional risk factors, suggesting that other factors may mediate the effects of these specific dietary habits on the risks for DR and DN. Moreover, after adjusting for glycated hemoglobin or LDL-c, the causal estimates of increased genetic susceptibility to DNP with pork intake remained statistically significant; in contrast, the output results after adjusting for the other four variables did not show significance. In addition, none of the *p*-values from the MVMR-Egger intercept tests indicated statistical significance, indicating the absence of horizontal pleiotropy (Supplementary Table S20).

## Discussion

The pathogenesis of DMCs is complex, which is cross-related to glucose and lipid metabolism disorders, inflammation, oxidative stress, impaired autophagy, gut microbiota imbalance and other factors (49–51). There is growing evidence that certain foods may increase or decrease the risk of DMCs in susceptible individuals (19, 52). As far as we are aware, this is the first large-scale MR study to characterize the causality between 17 dietary intakes and three DMCs. We observed higher genetically predicted salad/raw vegetable intake and fresh fruit intake were associated with an increased risk of DR and DN, while cereal intake had a protective effect against DR and DN. In addition, the reduced risk of DR was also associated with increased cheese consumption. Moreover, genetically predicted higher pork intake increased the risk of DNP. Furthermore, a MVMR analysis was conducted after adjusting for traditional risk factors of DMCs; the results indicated the potential independent risk factors. Our findings revealed that cheese, fresh fruit intake, salad/raw vegetable intake and pork intake possibly become an independent potential risk of DMCs occurrence. In contrast, the other three causal relationships identified in our study may be influenced by intermediate factors, indicating potential confounding.

Due to resistance, side effects and even toxicity of antidiabetic drugs, dietary therapy is a new direction for the treatment of diabetes (53). Cheese is a kind of fermented dairy product, which is integral part of human nutrition. It is considered as the carriers of proteins, calcium, fat, essential fatty acids, vitamins and phosphorus that are highly significant for physiological functions (54, 55). In addition, cheese contains a large number of lactic acid bacteria and metabolites, which is good for the balance of gut microbiota (56). Multiple meta-analyses and systematic reviews of the cohort studies point to a reduced risk of T2DM with cheese intake (57, 58). Our results were consistent with the conclusion from the previous studies. The results of a multicenter clinical study included a total of 8,122 participants were suggest that the risk of DR progression can be reduced by 40% in subjects who consume cheese ≥4 times per week (59). What’s more, the Mediterranean diet, which includes proportionally high intake of cheese, has been reported to protect against DR but not against DN (24), supporting our findings. Hence, cheese appears to be a protective factor for DR.

For fresh fruit, substantial uncertainties remain about its potential effects on risks of death and major vascular complications among those with diabetes. Fresh fruit intake might be a detrimental factor for DR in this MR study; this is not completely in line with previous observational studies. The main reason for this disagreement may be the difficulty in obtaining definitive causality in observational and cross-sectional studies due to confounding variables, including environmental and selection bias. In a large epidemiologic study of Chinese adults, higher consumption of fresh fruit was associated with significantly lower risk of diabetes and, among diabetic individuals, lower risks of death and development of major vascular complications (60). Similarly, a recent multi-state analysis of a prospective cohort revealed a protective effect of higher fresh fruit intake for primary and secondary prevention of T2DM (61). However, a meta-analysis showed no significant benefit of increasing fruit consumption on incidence of T2DM (62). A previous prospective study conducted in China found that higher fruit consumption during the second trimester was significantly associated with a higher risk of gestational diabetes mellitus (63). Coincidentally, Davison et al. (64) critically evaluate prospective cohort studies and RCTs, thinking fruit fiber gives little protection to T2DM, even for those at the upper limit of the range of intakes normally consumed. Findings from a pooled analysis of three large cohort studies showed that only some fruit types, such as blueberries, grapes/raisins, and apples/pears, but not other fruit types, had a significant protective effect on T2DM risk (65). Certain fruits are characterized by high glycemic load (GL) and low dietary fiber (66), which may also explain the association between fruits and T2DM risk found in this study. In addition, excessive fruit intake may lead to excessive fructose intake, which is associated with T2DM. A basic study found that short-term high-fructose diet can promote the increase of cytokines in monocytes and phagocytes in humans and mice, thereby causing an inflammatory response and diseases (67).

The effects of salads or raw vegetables on disease are less well studied. An earlier cross-sectional study found that frequent consumption of vegetables throughout the year was inversely associated with the risk of having undiagnosed non-insulin-dependent diabetes mellitus (NIDDM) (OR: 0.16, 95% CI: 0.04–0.69) (68). Alternatively, a cross-sectional study in Qingdao found that vegetable intake in women were inversely associated with T2DM prevalence (69). However, some studies have shown no relationship between vegetable intake and T2DM risk or levels of glycosylated hemoglobin (70–72). In our study, a genetically predisposed increase in salads or raw vegetables intake was causally associated with the risk of DN, indicating a potential risk factor. The high added sugar and trans unsaturated fatty acid bearing fats (trans fats) in salads may partly explain these results (73).

Pork is one of the red meats, often disparaged as risk factors for the development of diabetes in previous studies (74, 75). In a cross-controlled study involving 17 patients with T2DM and macroalbuminuria [24-h urinary albumin excretion rate (UAER) ≥ 200 microg/min], researchers found that withdrawing red meat from the diet reduced UAER (76). Rodrigues et al. also found that a red meat pattern was associated with a higher prevalence of diabetic nephropathy in a cross-sectional study of 329 outpatients with T2DM (77). However, in the MR analysis, pork, the representative of red meat, was not found to have a causal relationship with DN. Red meat (rich in carnitine and choline) significantly increased blood Trimethylamine N-oxide (TMAO) levels (78), while TMAO and its precursors (choline and L-carnitine) cause increase of fasting blood glucose and the enhancement of insulin resistance (79). On the other hand, pork contains high hemoglobin and iron, and its catalytic oxidation can destroy a variety of human components and cause oxidative stress damage. As mentioned earlier, the pathogenesis of DNP is related to oxidative stress. This may also partially explain the strong positive effect of pork in accelerating the risk of DNP progression in our analysis. Furthermore, Yan X and colleagues found no association between meat and the risk of DR progression (59). These findings are consistent with our results.

The study emphasizes the casualty of dietary in diabetes associated complications. The plausible mechanisms are as follows: First of all, diet can not only regulate various key molecules related to nutrition signal transduction, autophagy regulation, energy metabolism and other functions, but also effectively control inflammation and apoptosis. It can also improve glucose homeostasis and insulin secretion through *β*-cell regeneration (19). Second, abundant evidence indicates the effect of diet on the gut microbiota, focusing on the diet-microbiota crosstalk (80). Researchers recently conducted fecal microbiota transplantation experiments using inherited diabetic mice and found that mice transplanted with microbiota from DNP patients showed more severe peripheral neuropathy phenotype due to impaired intestinal barrier function, antigen load and systemic inflammatory response (81). And the “microbiota-gut-retina axis” also plays a critical role in the occurrence and development of DR. It has been found in animal experiments that the microbiota and its metabolites may promote retinal inflammation and barrier dysfunction, which may trigger DR (82).

Our research has some strengths. MR is a powerful statistical method for assessing possible causal relationships between underlying environmental factors and complex diseases. In order to address the bias issue, the GWAS data set with the most notable food intakes and diabetic complications participants were chosen. And exposures and outcomes were derived from European populations. However, several potential limitations should be observed. First, the inclusion of a European ancestries may limit the generalizability of our findings. The causal relationship between food intakes and diabetic microvascular complications needs to be further studied in more pedigree populations. Second, the demographic characteristics, clinical information, food dosage and dietary combinations were not available in the GWAS database, and subgroup analysis could not be performed due to insufficient data. Deeper and finer whole-genome sequencing is needed to better characterize the causal relationship between fresh fruit, pork and cheese intake and DMCs. At the same time, we look forward to conducting prospective, multi-center, large-sample, randomized controlled trials with longer follow-up time to clarify this association as soon as possible, and to realize individualized treatment and precision medicine of dietary therapy for diabetic microangiopathy.

## Conclusion

In summary, we draw conclusions from a limited perspective that cheese intake has potential preventive value for DR, whereas fresh fruit intake, salad/raw vegetable intake and pork intake may be independent risk factors for DR, DN and DNP, respectively.

## Data Availability

The original contributions presented in the study are included in the article/Supplementary material, further inquiries can be directed to the corresponding author.

## Footnotes

^1^ https://gwasmrcieu.ac.uk/datasets

^2^ https://biobank.ctsu.ox.ac.uk/crystal/label.cgi?id=100052

^3^ https://r5.finngen.fi/

## Supplementary material

SUPPLEMENTARY FIGURE S1 The symmetry of funnel plots indicated a balanced pleiotropy; (A) is for cheese intake on DR; (B) is for salad/raw vegetable intake on DR; (C) is for fresh fruit intake on DR; (D) is for cereal intake on DR; (E) is for Salad/raw vegetable intake on DN; (F) is for fresh fruit intake on DN; (G) is for cereal intake on DN; (H) is for pork intake on DNP.

SUPPLEMENTARY FIGURE S2 Leave-one-out plots demonstrated no individual SNPs that could significantly affect the causal effects; (A) is for cheese intake on DR; (B) is for salad/raw vegetable intake on DR; (C) is for fresh fruit intake on DR; (D) is for cereal intake on DR; (E) is for Salad/raw vegetable intake on DN; (F) is for fresh fruit intake on DN; (G) is for cereal intake on DN; (H) is for pork intake on DNP.
