# Supplementary figures and images for: Dietary patterns and diabetic microvascular complications risk: a Mendelian randomization study of European ancestry
Source: Front Nutr. 2024 Nov 1;11:1429603. doi: 10.3389/fnut.2024.1429603 (PMC11566142; doi:10.3389/fnut.2024.1429603)

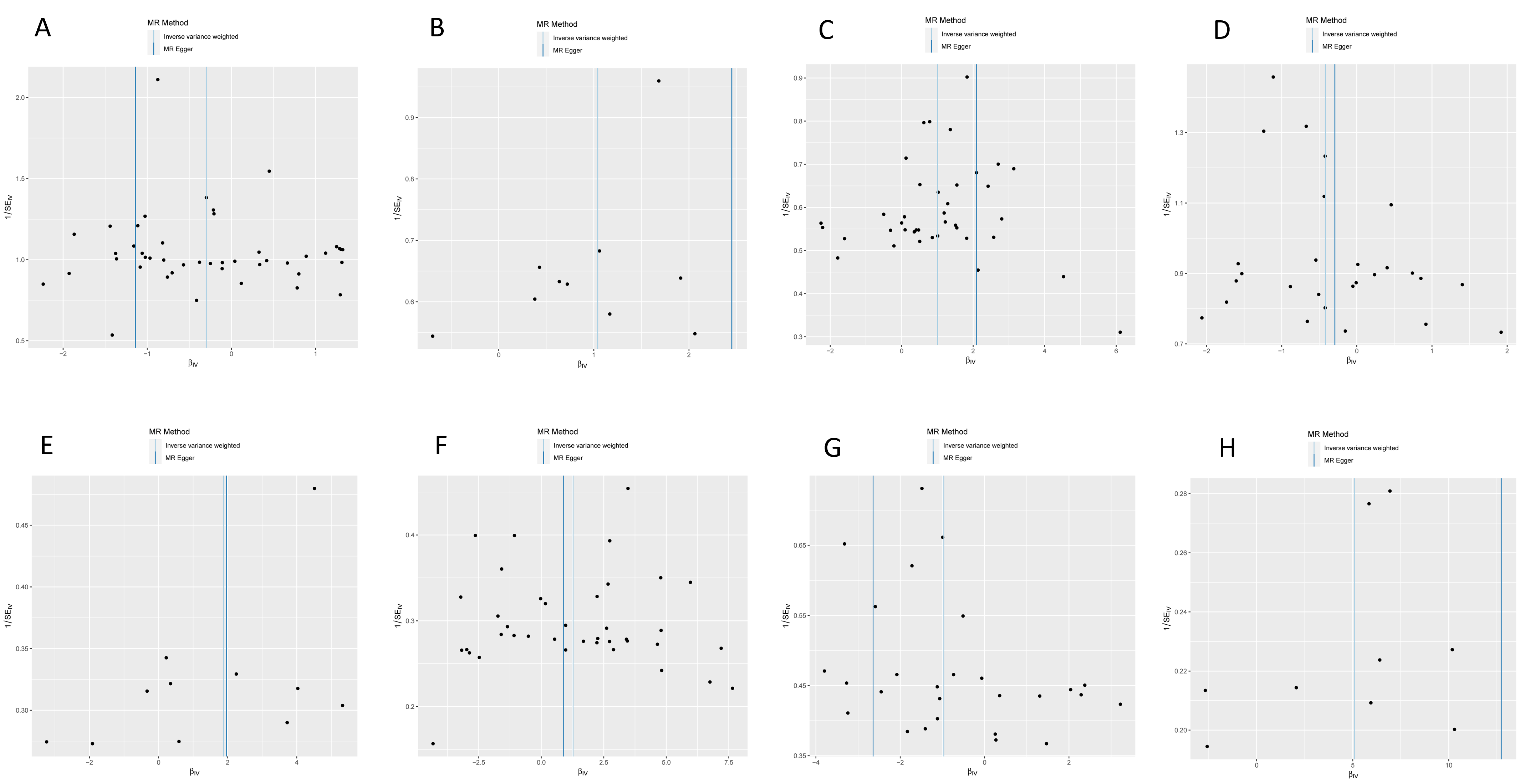

Supplement: SUPPLEMENTARY FIGURE S1 — The symmetry of funnel plots indicated a balanced pleiotropy; (A) is for cheese intake on DR; (B) is for salad/raw vegetable intake on DR; (C) is for fresh fruit intake on DR; (D) is for cereal intake on DR; (E) is for Salad/raw vegetable intake on DN; (F) is for fresh fruit intake on DN; (G) is for cereal intake on DN; (H) is for pork intake on DNP. [file Image_1.TIF]

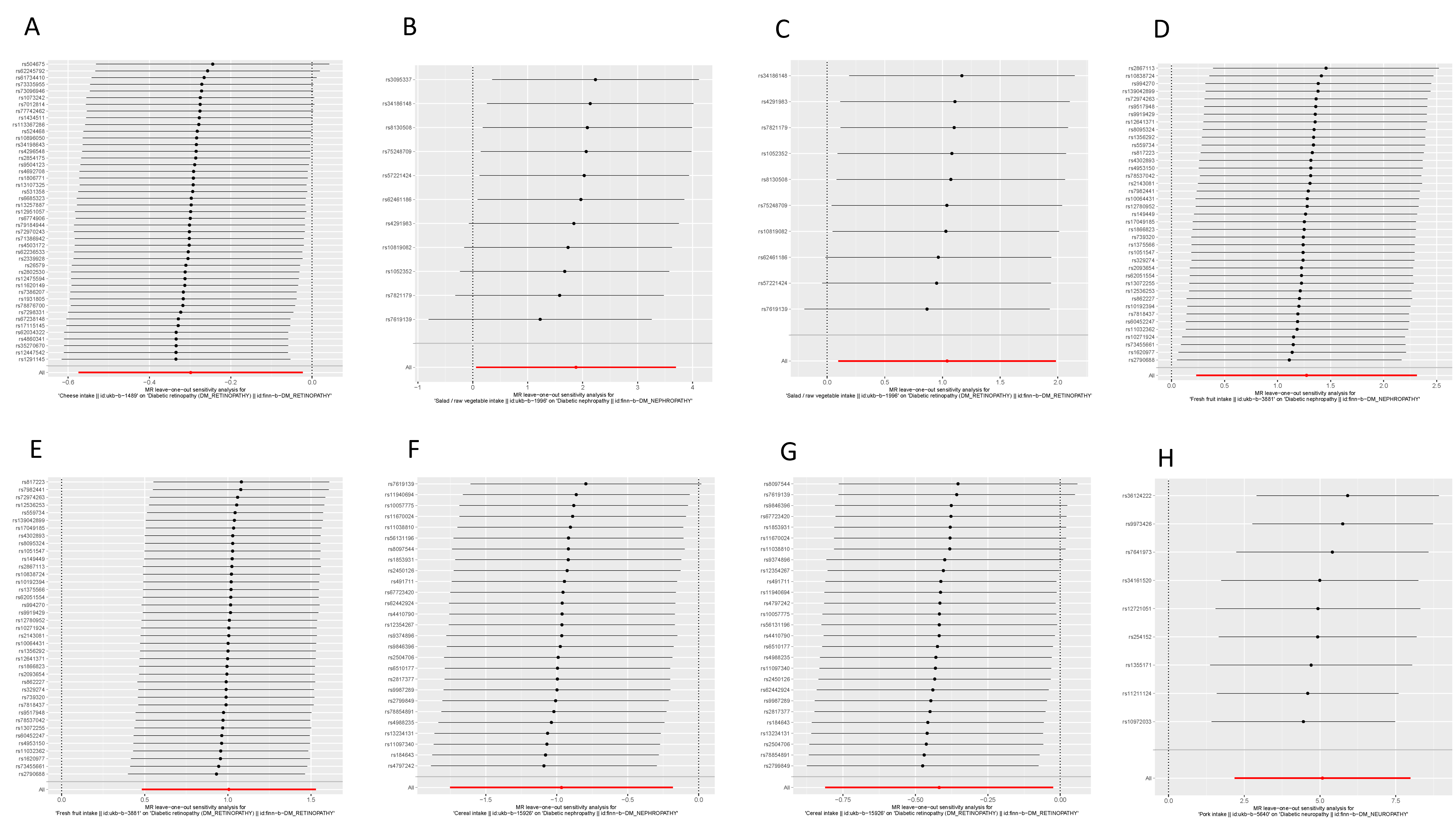

Supplement: SUPPLEMENTARY FIGURE S2 — Leave-one-out plots demonstrated no individual SNPs that could significantly affect the causal effects; (A) is for cheese intake on DR; (B) is for salad/raw vegetable intake on DR; (C) is for fresh fruit intake on DR; (D) is for cereal intake on DR; (E) is for Salad/raw vegetable intake on DN; (F) is for fresh fruit intake on DN; (G) is for cereal intake on DN; (H) is for pork intake on DNP. [file Image_2.TIF]
